# Atomic number dependence of *Z* contrast in scanning transmission electron microscopy

**DOI:** 10.1038/s41598-018-30941-5

**Published:** 2018-08-17

**Authors:** Shunsuke Yamashita, Jun Kikkawa, Keiichi Yanagisawa, Takuro Nagai, Kazuo Ishizuka, Koji Kimoto

**Affiliations:** 1 National Institute for Materials Science, 1-1 Namiki, Tsukuba, Ibaraki, 305-0044 Japan; 2 HREM Research Inc., 14-48 Matsukazedai, Higashimatsuyama, Saitama, 355-0055 Japan

## Abstract

Annular dark-field (ADF) imaging by scanning transmission electron microscopy (STEM) is a common technique for material characterization with high spatial resolution. It has been reported that ADF signal is proportional to the *n*th power of the atomic number *Z*, *i*.*e*., the *Z* contrast in textbooks and papers. Here we first demonstrate the deviation from the power-law model by quantitative experiments of a few 2D materials (graphene, MoS_2_ and WS_2_ monolayers). Then we elucidate ADF signal of single atoms using simulations to clarify the cause of the deviation. Two major causes of the deviation from the power-law model will be pointed out. The present study provides a practical guideline for the usage of the conventional power-law model for ADF imaging.

## Introduction

Annular dark-field (ADF) imaging by scanning transmission electron microscopy (STEM)^1–3^ is a common technique for material characterization with high spatial resolution, and it has been applied to various materials, such as oxides^4–6^, catalysts^7,8^, atomic clusters^9^, dopants^10,11^, quasicrystals^12^ and two-dimensional (2D) materials^13^. Major advantages of ADF imaging are its intuitive contrast and atomic sensitivity. The atomic number dependence of ADF signal has been investigated since a pioneering study by Crewe *et al*.^3^, and it has been reported that ADF signal is proportional to the *n*th power of the atomic number *Z*, *i*.*e*., the *Z* contrast in textbooks^14,15^ and papers^13,16,17^. It should be noted that the original *Z* contrast proposed by Crewe and ADF imaging are technically different, and a historical review about *Z* contrast techniques is given by Treacy^17^.

ADF signal can be quantitatively analyzed as the scattering probability of incident electrons^18^, *i*.*e*., the ratio (*I*_ADF_/*I*_0_) of the current measured using an ADF detector *I*_ADF_ to the incident probe current *I*_0_; we call the ratio ‘ADF contrast’ *Q*_ADF_ [%] in this paper. Because the current measured using an ADF detector is low (*e*.*g*., less than 0.1 pA^19^ for a monolayer graphene), quantitative ADF imaging is technically challenging, particularly for 2D materials. In our previous studies, we established quantitative ADF imaging, in which the nonlinear response of the ADF detection system was corrected. The experimental ADF contrast of a monolayer graphene and a simulated image well agreed within the quantum noise level (see Fig. S2 of the Supplementary Information or our previous work^20^). Two-dimensional materials are ideal standard specimens that have uniform thickness and atomic structures. Note that common STEM specimens prepared by conventional techniques (*e*.*g*., ion-beam thinning) are covered by a surface damage layer, whose thickness is unknown, preventing accurate analyses that are free from fitting parameters.

In this study we reexamine the atomic number dependence of ADF signal by performing experiments and simulations. We first demonstrate the deviation from the power-law model by the quantitative ADF imaging of monolayer 2D materials. Then we elucidate ADF signal of single atoms using kinematical calculation and phase-object simulation. Two major causes of the deviation from the power-law model will be pointed out; the nonmonotonic *Z* dependence of the atomic radius and the dynamical diffraction of a single atom.

## Results and Discussions

### Experiment of quantitative ADF imaging of monolayer 2D materials

We measured the ADF contrast of monolayer 2D materials (graphene, MoS_2_ and WS_2_) using an aberration-corrected STEM instrument (FEI, Titan cubed) at an acceleration voltage of 80 kV under three different camera-length conditions: (i) 145, (ii) 115 and (iii) 91 mm, *i*.*e*., ADF imaging were performed with three ADF detection angle ranges. The inner angle in ADF signal detection was limited by an ADF detector (E.A. Fischione Instruments, Inc., Model 3000), although the outer angle was limited by the microscope diaphragm to *β*_outer_ = 200 mrad. Further experimental details are given in Methods and the Supplementary Information.

**Figure 1:**
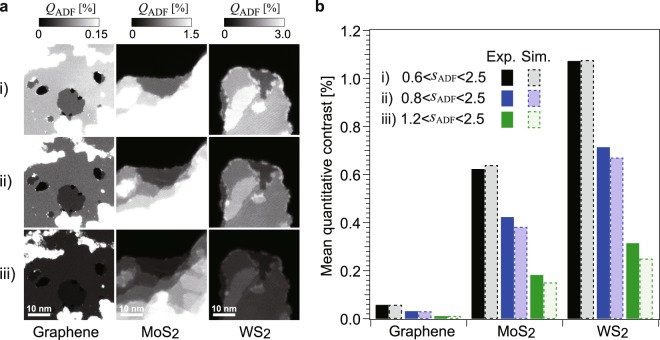
Experimental ADF images of 2D materials and spatially averaged ADF contrast of each monolayer. (**a**) ADF images of the 2D materials (graphene, MoS_2_ and WS_2_) observed under three camera lengths, (i) 145 mm, (ii) 115 mm and (iii) 91 mm at an acceleration voltage of 80 kV. The experimental images show the ADF contrast *Q*_ADF_ [%], which corresponds to the scattering probability of incident electrons. (**b**) Spatially averaged ADF contrast of each monolayer under the three experimental conditions. Experimental results and the results of phase-object simulations are shown by bars with solid and broken lines, respectively. The ADF detection angle ranges *s*_ADF_ for the three conditions are given as the scattering parameter *s* (=***sinθ***/***λ***) [Å^−1^].

Quantitative ADF images of the 2D materials and the averaged ADF contrast of each monolayer are shown in Fig. 1a,b, respectively. Although the microscope can resolve individual atoms, here we discuss the spatially averaged ADF contrast, which is experimentally reproducible because it is independent of the objective lens settings such as geometrical aberrations. The applied ADF imaging conditions (i), (ii) and (iii) are typical for various applications; the inner ADF angles *β*_inner_ for the three camera lengths are (i) 48.4, (ii) 62.3 and (iii) 96.9 mrad. These inner angles respectively correspond to scattering parameters *s*_inner_ of (i) 0.58, (ii) 0.75 and (iii) 1.2 Å^−1^, where *s* is the scattering parameter defined as *s* = sin*θ*/*λ*.

We evaluate the parameter *n* in the power law *Z*^*n*^ on the basis of these experimental results. Since the few 2D materials are not monoatomic and their atomic densities are not equal, we calculate the parameter *n* as

1 $${Q}_{{\rm{ADF}}}\propto {S}^{-1}\sum _{i}{Z}_{i}^{n},$$

where *S* is the projected area of a unit cell and *Z*_*i*_ is the atomic number of the *i*th atom within the unit cell. As shown in Table 1, the parameter *n* is calculated under three evaluation conditions, which are parameter fittings using (a) the three specimens, (b) graphene and MoS_2_, and (c) MoS_2_ and WS_2_. It is found that the power-law model cannot fully represent the experimental data. For instance, the *Z* dependence for the case of graphene and MoS_2_ is *n* = 1.69–1.90, as shown in column (b) of Table 1; however, the value for the case of MoS_2_ and WS_2_ is found to be *n* = 1.30–1.33 under the three experimental conditions, as shown in column c). Thus, the experimental results do not follow the power-law model. It should also be noted that phase-object simulations (bars with broken lines in Fig. 1b) reproduce these experimental results; therefore, the power-law model does not hold in both the quantitative experiments and the phase-object simulations. Now the central theme of this paper is to clarify the reason for this breakdown by evaluating cross sections of single atoms. We calculate the scattering cross sections using two approaches: kinematical calculation and phase-object simulation.

**Table 1:** Parameter *n* calculated from experimental results in Fig. 1b.

Experimental settings ADF inner angle (mrad)	(a) Based on three specimens	(b) Based on graphene & MoS_2_	(c) Based on MoS_2_ & WS_2_
(i) 48.4	1.34	1.69	1.33
(ii) 62.3	1.33	1.74	1.30
(iii) 96.9	1.37	1.90	1.33

### Kinematical calculation of single atoms

We first apply a kinematical approach to calculate the ADF signal intensity of a single atom. Under the kinematical approximation (*i*.*e*., the first Born approximation), the ADF scattering intensity is equal to the integrated intensity, which is the square of the amplitude of the atomic scattering factor *f* from *β*_*inner*_ to *β*_*outer*_ as follows:

2 $$({\rm{ADF}}\,{\rm{scattering}}\,{\rm{intensity}})={\int }_{{\beta }_{inner}}^{{\beta }_{outer}}\,2\pi \beta \,f{(\beta )}^{2}d\beta .$$

**Figure 2:**
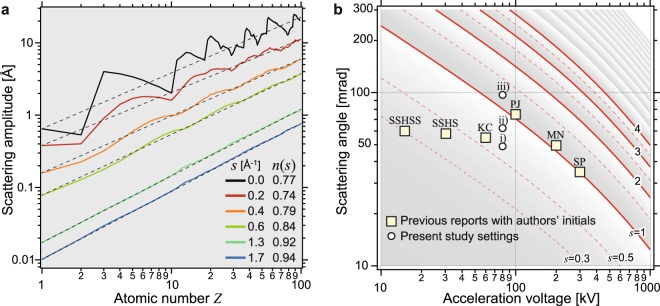
Atomic number dependence of atomic scattering factors in log-log plot and experimental settings for ADF imaging in pioneering studies. (**a**) Amplitudes of atomic scattering factors for different scattering parameters *s* (=**sin*θ***/***λ***). Broken lines are power-law fittings *Z*^*n*(*s*)^ for each value of scattering parameter. (**b**) Scattering angle as a function of acceleration voltage and scattering parameter. Marks show ADF inner angles of the present study and the published papers as follows: SP^11^, MN^5^, PJ^2^, KC^13^, SSHS^24^ and SSHSS^25^.

Because the ADF detector covers up to a high scattering angle (*e*.*g*., *β*_outer_ = 200 mrad, corresponding to *s*_outer_ = 2.5 [Å^−1^] at 80 kV), atomic scattering factors should be valid up to a high scattering angle. We use the atomic scattering factors published by Weickenmeier and Kohl^21^, although another series of atomic scattering factors by Kirkland^14^ gives similar results. Since the amplitude of the atomic scattering factors rapidly decreases as a function of the scattering angle, the *Z* dependence of ADF contrast is mainly characterized by the inner ADF angle. Figure 2a shows the scattering amplitude *f*(*s*) for various scattering parameters *s* as a function of atomic number *Z*, where both axes have a logarithmic scale. In the case of low scattering parameters (*s* < 1), the amplitudes do not monotonically increase, and this irregular feature is related to the *Z* dependence of the atomic radius. In contrast, the *Z* dependence at high scattering parameters (*s* > 1) becomes straight in the log-log plot, and the slope *n* of *Z*^*n*^ is close to one as tabulated in Fig. 2a. Since the ADF scattering intensity is the square of the amplitude under the kinematical approximation, the linear *Z* dependence (*n* = 1) of the amplitude corresponds to the *Z*^2^ dependence of ADF contrast. High-angle scattering is considered to be a small impact parameter and to be less sensitive to chemical bonding or the atomic radius. Although a high angle was originally proposed to reduce the elastic Bragg diffraction^2,22^ of crystalline specimens, high-angle detection is also effective for deducing unscreened Rutherford scattering even in the case of a single atom.

In diffraction physics for crystallography, the atomic scattering factor is given as a function of the scattering parameter *s* [Å^−1^]^23^, and the actual scattering angle *β* [rad] should be converted to a scattering parameter *s* [Å^−1^] using the acceleration voltage *E*_0_ [V] as

3 $$s({E}_{0},\beta )=\frac{\beta {E}_{0}^{1/2}(1+0.9785\times {10}^{-6}{E}_{0})}{24.52}.$$

We have surveyed experimental ADF settings in several pioneering studies as shown in Fig. 2b. Although Pennycook and Jesson reported the use of high-angle ADF (HAADF) imaging^2^ to realize incoherent imaging, where their ADF inner angle corresponded to *s*_inner_ ~ 1 Å^−1^, recent STEM experiments have often been performed at a low acceleration voltage^24–26^ to reduce knock-on damage and at a relatively small ADF inner angle (*e*.*g*., *s*_inner_ < 1 Å^−1^) to improve detection efficiency^13,27^. Aberration-corrected STEM^28^ allows us to visualize atomic structures at a lower acceleration voltage, resulting in a low scattering parameter (*s*_inner_ < 1) in ADF imaging. Then, the simple power-law *Z* dependence is no longer applicable in the case of a small scattering parameter.

The above-mentioned kinematical approach is an approximation in which the plane wave of an electron is weakly scattered. Incident electrons at a lower acceleration voltage are actually scattered strongly and the kinematical approximation might no longer be valid even in the case of single atoms. As shown in Fig. 1b and Table 1, the ADF contrast obtained for a high scattering parameter, iii) *s*_inner_ = 1.2, also shows the breakdown of the power-law model; this suggests that another factor causes the breakdown of the power-law model.

### Phase-object simulation of single atoms

Next we investigate the ADF signal intensity by performing phase-object simulations, which are generally used as multislice simulations in electron microscopy^14,29^. The profiles of atomic potentials for electron scattering are generated using the Fourier transforms of atomic scattering factors, and a coherently focused incident probe is scanned across the atomic potentials, which are placed at the center of a 2.02 × 2.02 nm cell in this study. The intensity of elastic scattering in the ADF detection range *s*_ADF_ is integrated, and then an ADF image is constructed. The cross section of each atom is evaluated as the integrated scattering intensity of the ADF image. Since the atomic potentials are sharp in real space (see the Supplementary Information), simulations that include a high frequency of up to *s* = 25 [Å^−1^] in reciprocal space must be performed. The convergence semiangle of the incident probe is set to a scattering parameter *s*_c_ of 0.25 Å^−1^ (*e*.*g*., 30 mrad at 40 kV), which was used for all the acceleration voltage settings of 40, 80, 300 and 800 kV. Although the interaction between an incident electron and an atomic potential depends on the acceleration voltage (*i*.*e*., interaction parameter), the incident probe profile is the same because of the same convergence setting *s*_*c*_. The incident probe was scanned with a sufficiently fine step of 0.2 Å. To minimize the above-mentioned nonmonotonic *Z* dependence of the atomic scattering factor, the ADF detection range is set to a high angle of 1.66 < *s*_ADF_ < 2.49 [Å^−1^], which is, for example, 200 to 300 mrad at 40 kV.

**Figure 3:**
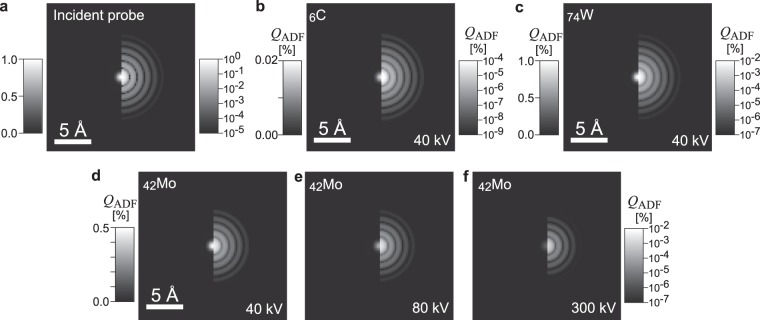
Incident probe and ADF images of various single atoms obtained by phase-object simulation. (**a**) Incident probe and (**b**–**f**) ADF images simulated under various conditions in linear (left) and logarithmic (right) brightness scales. The ADF detection angles are set to high scattering parameter of 1.66 < *s*_ADF_ < 2.49 [Å^−1^]. No aberration of the objective lens is implemented.

Figure 3a shows the incident probe in the simulation in both linear (left) and logarithmic (right) brightness scales, in which the peak intensity is normalized to one. The full width at half maximum (FWHM) of the incident probe is 1.1 Å, and weak concentric Airy fringes are observed. Figure 3b–f are ADF images of single atoms simulated under various conditions. The basic feature of the ADF images (Fig. 3b–f) is very similar to the incident probe profile; the FWHMs of the atom profiles are 1.1 Å and they also exhibit Airy fringes. The features in the ADF images are identical at the different acceleration voltages as shown in Fig. 3d–f. These simulations are well represented by the incoherent imaging approximation, in which an observed image corresponds to the convolution between the incident probe and the object function.

**Figure 4:**
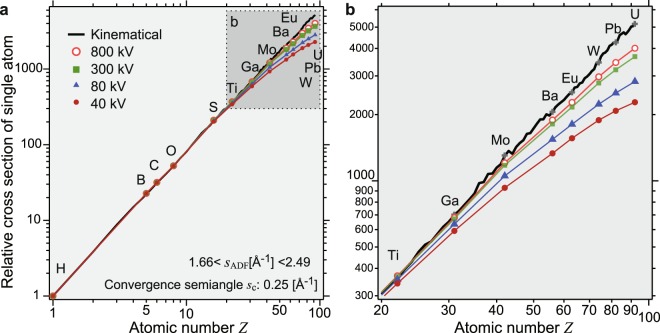
Atomic number dependence of integrated ADF contrast of single atoms. (**a**) *Z* dependence of the integrated ADF contrast, which is normalized by that of a hydrogen atom and (**b**) enlarged graph. Marks show the results of phase-object simulations and the black line represents the kinematical calculation. Both axes have a logarithmic scale. Each cross section of the elements is normalized by that of a hydrogen atom. The ADF detection angle range is 1.66 < *s*_ADF_ < 2.49 [Å^−1^]. The convergence semiangle in the phase-object simulation is *s*_c_ = 0.25 [Å^−1^]. Note the breakdown of the power-law model, *i*.*e*., deviation from the straight line in the log-log graph, particularly for heavy elements, at lower acceleration voltages.

We evaluate the *Z* dependence of the ADF contrast by performing phase-object simulations of thirteen elements for four different acceleration voltages, as shown in a log-log plot (Fig. 4a). The ADF cross sections for the elements are normalized by that of a hydrogen atom. The kinematical calculation is plotted as a black solid line for all elements. The almost straight line representing the kinematical calculation in the log-log plot indicates the validity of the power-law model at such a high ADF inner angle. However, the phase-object simulation shows that there is a systematic deviation, particularly for high-*Z* elements, at a lower acceleration voltage. The deviation becomes evident for many elements (*Z* > 20) as shown in Fig. 4b. We confirmed the same deviation even under very high outer-angle condition (Fig. S5a). This is due to the coherent strong scattering of incident electrons, *i*.*e*., dynamical scattering by a single atom. It should be noted that Cowley pointed out the failure of kinematical approximation for scattering from single heavy atoms^30^. Even in the case of ADF imaging, the atomic scattering potentials are coherently illuminated and the phase change of the incident electron wave at the center of the atomic scattering potential is large, resulting in the decrease in intensities at high scattering angle compared with the kinematical approximation. By contrast, the dynamical scattering enhances the intensity at lower angle as described in the Supplementary Information (Fig. S5b). These results can explain the above-mentioned difference in the *Z* dependences of graphene-MoS_2_ and MoS_2_-WS_2_.

## Conclusion

In this study we have revealed the breakdown of the power-law model in STEM ADF imaging through experiments and simulations. The deviation from the power-law model becomes evident at a small ADF detection angle and for an atom with a relatively high *Z* (*e*.*g*., *Z* > 20). In conclusion the two major causes of the deviation from the power-law model are the nonmonotonic *Z* dependence of the atomic radius and the dynamical diffraction of a single atom, and such breakdown often occurs in modern low-voltage electron microscopy. Although the deviation is not negligible even at a high acceleration voltage (*e*.*g*., 300 kV) and high scattering angle, quantitative analysis can be performed on the basis of the accurate phase-object simulations. The present study provides guidelines for the usage of the conventional power-law model for ADF imaging, which is still an excellent approximation for light elements in the case of high-angle ADF imaging.

## Materials and Methods

### Specimen preparation and STEM observation

A chemical-vapor-deposited graphene (TEM2000GL, ALLIANCE Biosystems), which was transferred onto a Cu mesh without a supporting film, was used as a graphene specimen. Solutions of WS_2_ and MoS_2_ (Graphene Supermarket) were dispersed on holey carbon films.

Experimental ADF images were obtained using an aberration-corrected STEM instrument (FEI, Titan cubed) at an acceleration voltage of 80 kV. An ADF detector (E.A. Fischione Instruments, Inc., Model 3000) and analog-digital (AD) converter (Gatan, DigiScan II) were used to acquire the ADF images. The ADF images are processed using software (Gatan, DigitalMicrograph), in which the nonlinear responses of the ADF detector and AD converter were corrected using an empirical function. The incident probe current was 20 pA, which was measured using a charge-coupled device (CCD) camera whose sensitivity was calibrated in advance. The ADF angle range should be carefully evaluated^31,32^ and our procedure was given in the Supplementary Information.

To avoid contamination during observations, STEM specimens were annealed in vacuum at 523 K before STEM observations. The specimen temperature during the STEM observations was room temperature for graphene and 873 K for MoS_2_ and WS_2_. A specimen heating holder (Protochips, Aduro) was used for the MoS_2_ and WS_2_ specimens.

### Kinematical calculation and phase-object simulation of ADF images

Kinematical calculations were performed using the atomic scattering factors published by Weickenmeier and Kohl^21^, in which the factors are expressed with nine parameters, whose equation is given in the Supplementary Information. In the phase-object simulation, ADF images are simulated using a multislice software program (HREM Research Inc., xHREM and STEM plug-in). The simulations were performed up to a high frequency of *s* = 25 [Å^−1^] and the scanning step was 0.2 Å. Further technical details are described in the Supplementary Information.

## Electronic supplementary material

Supplementary Information

## Data Availability

The datasets generated during the current study are available from the corresponding author on reasonable request.
