# Supplementary material for: Atomic number dependence of Z contrast in scanning transmission electron microscopy
Source: Sci Rep. 2018 Aug 17;8:12325. doi: 10.1038/s41598-018-30941-5 (PMC6098135; doi:10.1038/s41598-018-30941-5)
Supplement: Supplementary file 1 — Supplementary Information [file 41598_2018_30941_MOESM1_ESM.pdf]

Supplementary Information

Atomic number dependence of *Z* contrast in scanning transmission electron microscopy

S. YAMASHITA[1], J. KIKKAWA[1], K. YANAGISAWA[1], T. NAGAI[1], K. ISHIZUKA[2] and K. KIMOTO[1]
[1]National Institute for Materials Science, 1-1 Namiki, Tsukuba, Ibaraki 305-0044, Japan
[2]HREM Research Inc., 14-48 Matsukazedai, Higashimatsuyama, Saitama 355-0055, Japan
Correspondence should be addressed to K.K. (email: kimoto.koji@nims.go.jp)

Contents

1. Experimental details of quantitative annular dark-field (ADF) imaging by scanning transmission electron microscopy (STEM)
2. Atomic scattering factors for electrons
3. Phase-object simulations of ADF imaging
4. Effect of detector angle on *Z* dependence

1. Experimental details of quantitative annular dark-field (ADF) imaging by scanning transmission electron microscopy (STEM)

We used an electron microscope (FEI, Titan cubed) equipped with double spherical-aberration correctors (CEOS, DCOR and CETCOR) operated at an acceleration voltage of 80 kV. An ADF detector (E.A. Fischione Instruments, Inc., Model 3000) and an analog-to-digital (AD) converter (Gatan, DigiScan II) were used. A single electron can be detected using the present system. The observed ADF images were processed using DigitalMicrograph software (Gatan).

The ADF contrast Q_{ADF} corresponds to the probability of scattering to ADF detector angle range. The observed ADF signal must be converted to quantitative ADF contrast $Q_{\mathrm{ADF}}=I_{\mathrm{ADF}}/I_0$, where I_{ADF} and I_0 are the ADF detector current and incident probe current, respectively. We converted the observed ADF signal to the ADF detector current using an empirical function, in which the correction of the nonlinear response (Fig. S1) of the detection system is implemented[1]. The incident beam current of 20 pA was measured using a charge-coupled device (CCD) camera whose sensitivity (*i.e.*, conversion efficiency) was calibrated in advance; a single electron generated 10.3 CCD counts at 80 kV.

The ADF detection angle range is determined by the ADF detector and electron microscope optics. The actual inner angle of the ADF detector was measured by scanning the incident electron on the detector[1]. Although the diameter of the ADF detector is sufficiently large, the maximum detectable angle is limited by the diaphragm of the electron microscope. We experimentally measured the maximum ADF detector angle, and it was found to be 200 mrad, which is smaller than that estimated by mechanical dimensions of the ADF detector (see Appendix of our previous paper[1]). Recently, precise assessment of ADF detectors has also been reported by Jones *et al* [2,3].

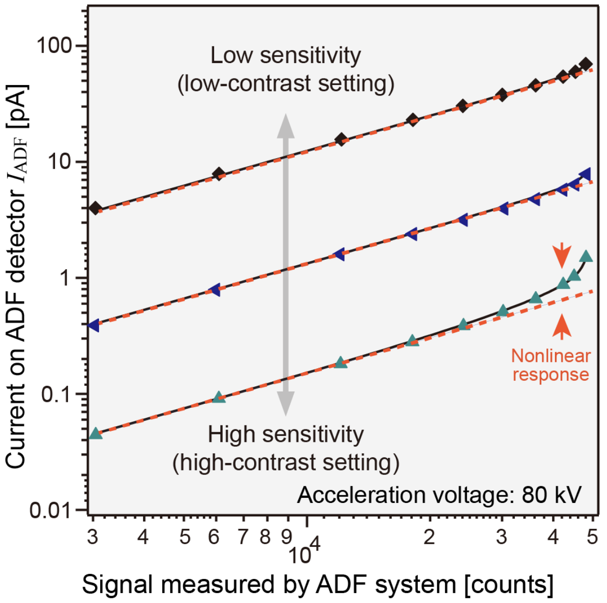

Figure S1. Nonlinear response of ADF detection system measured at 80 kV.
The sensitivity of the ADF detection system depends on the contrast setting of the system. Marks are measured responses, solid lines are empirical fittings and broken lines are linear relations. A high-contrast setting is used for high-sensitivity observations of 2D materials, in which the nonlinear response becomes evident.

An example of experimental ADF images is shown in Fig. S2a. Here the specimen is a monolayer graphene and the experimental conditions are as an acceleration voltage of 80 kV, a probe current of 26 pA, and an ADF inner angle of 48 mrad. A multiple fast acquisition technique[4,5] was used to improve the signal-to-noise ratio. We also performed a phase-object simulation (Fig. S2b), in which the diameter of the effective source distribution was assumed to be 80 pm in full width at half maximum. Note that the average intensity does not depend on the effective source distribution because the effect of a source distribution is theoretically equal to a simple convolution. We also simulate quantum noise based on the Poisson distribution of the number of electrons at each pixel, and the simulation and experimental results are in good agreement within the quantum noise level as shown in Fig. S2[1,6].

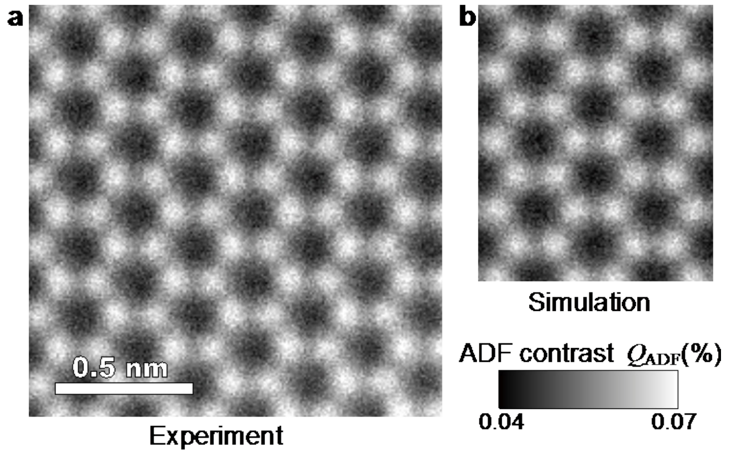

Figure S2. Quantitative ADF contrast of monolayer graphene.
(a) Experimental and **(b)** simulated images. In the simulation, quantum noise based on the Poisson distribution of the number of electrons is implemented.

2. Atomic scattering factors for electrons

We used the atomic scattering factors (atomic form factors) for electrons $f(s)$ published by Weickenmeier and Kohl[7]. The atomic scattering factors are parameterized by the following equation:

$$f(s) = s^{-2} \sum_{i=1}^{6} A_i[1 - \exp(-B_i \cdot s^2)],$$

$$A_1 = \frac{0.02395\,Z}{3(1+V)},\ A_1 = A_2 = A_3,\ A_4 = VA_1,\ A_4 = A_5 = A_6,\ s = \sin\theta/\lambda \qquad \text{(S1)}$$

where A_1, A_2, B_1, B_2, B_3, B_4, B_5, B_6 and V are parameters given in their paper[7]. The hydrogen atomic scattering factor is separately given in the paper. We also used the atomic scattering factors reported by E.J. Kirkland[8], which were almost equal to those reported by Weickenmeier and Kohl.

3. Phase-object simulations of ADF imaging

Phase-object simulations for electron microscopy are generally used as multislice simulations, in which an atom is regarded as a phase object to an electron wave. In the phase-object simulation for ADF imaging a convergent incident probe is scanned across a phase object, and elastic scattering intensities on the ADF detector range are integrated. The phase-object simulations in the present study were performed using a multislice calculation software package (HREM Research Inc., xHREM and STEM plug-in). The atomic scattering potential deduced from the corresponding atomic scattering factor has a very confined profile at the origin as shown in Fig. S3. To simulate such a high-frequency profile the simulation must be performed with a wide reciprocal space whose highest scattering vector is $s=\sin\theta/\lambda=25$ [$Å^{-1}$]. Bird and King[9] pointed out that the absorptive form factor, which is a fundamental parameter used to calculate ADF scattering, should be calculated up to a high angle of s~30 [$Å^{-1}$].

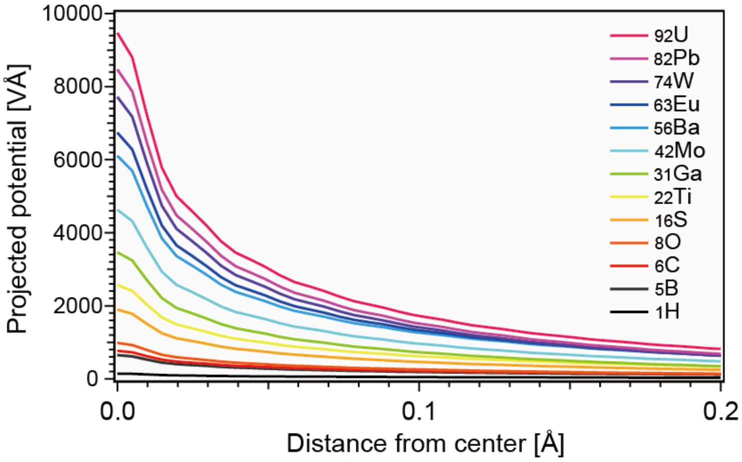

Figure S3. Projected atomic potential profiles of various atoms calculated using the atomic form factors of Eqn. (S1).

There are two major approaches to calculate ADF images in phase-object simulations: frozen-phonon approximation and thermal-diffuse-scattering (TDS) absorption-potential approximation. Here the purpose of the simulation is to calculate the integrated scattering intensity from a single atom; therefore, we calculated the elastic scattering of a single atom without TDS absorption, which is similar to the frozen-phonon approximation with a single configuration. The ADF signals scattered using atomic potentials (Fig. S3) are simulated.

Figure S4 shows incident probe profiles at different convergence settings without geometrical aberrations. Although the wavelength of an electron wave depends on the acceleration voltage, the incident electron profile does not depend on the acceleration voltage because the same convergence setting of s_c=0.25 [Å^{-1}] was used in this study. The full width at half maximum of the profile under the convergence setting of s_c=0.25 [Å^{-1}] is 1.1 Å. The atomic potential of a single atom is placed at the center of a 20.2 Å squared area, and the incident probe is scanned with steps of 0.2 Å. The integrated intensities over the square area are evaluated.

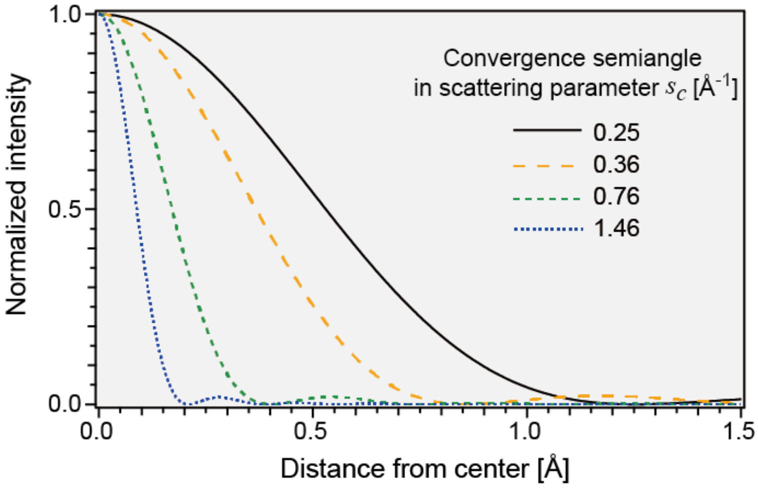

Figure S4. Incident probe profiles at various convergence settings with no geometrical aberrations of the objective lens. In the phase-object simulation, the setting s_c=0.25 (black solid line) is applied.

4. Effect of detector angle on *Z* dependence

We investigated *Z* dependence of ADF imaging at a detection range of $1.66<s_{ADF}<2.49$ [Å^{-1}], as shown in Fig. 4. We also investigated *Z* dependence for a very-high outer-angle. Figure S5 shows phase-object simulations of thirteen elements at 80 kV. It is found from Fig. S5a that the deviation from the power-law is evident even for the case of the very-high outer-angle (1000 mrad). By contrast, intensity in lower angle (0-50 mrad) for heavy elements becomes high in comparison with the power law fitted to the light elements (Z<60) (see triangles of Fig. S5b). Because detection probability simulated up to 1000 mrad (open squares of Fig. S5b) are almost one, we can neglect intensity scattered at more than 1000 mrad. The deviation from the power-law of high angle (50-1000 mrad) scattering is almost equal to that of low angle (0-50 mrad) scattering (see two arrows of Fig. S5). This means that the effect of dynamical scattering by heavy atoms is the confinement of transmitted electrons to a **lower** scattering angle.

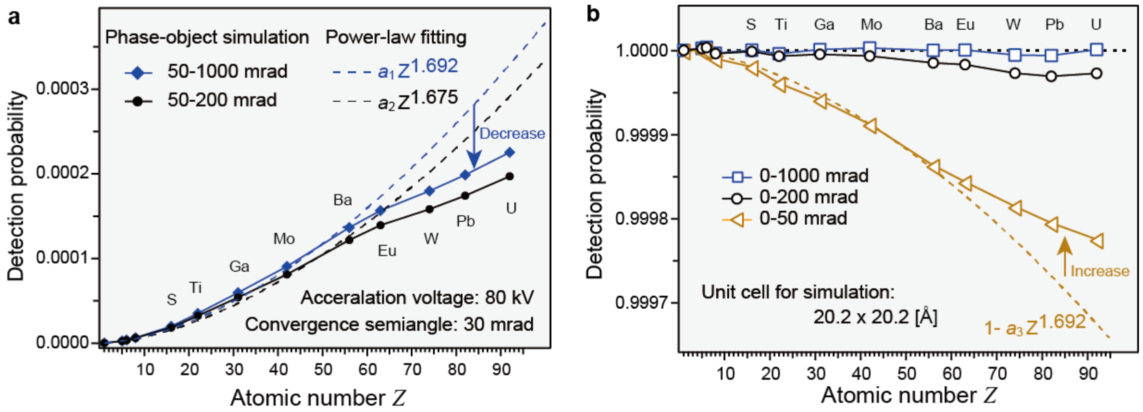

Figure S5. Detection probability obtained by phase-object simulations of single atoms at 80 kV. **(a)** Atomic number dependence of detection probability for two ADF angle ranges; 50-200 mrad and 50-1000 mrad. **(b)** Atomic number depencence of detection probability including low scattering angle. Low-angle (0-50 mrad) intensity (open triangles) deviates from the power law for heavy atoms. Broken lines are power-law fittings, in which a_1, a_2 and a_3 are constants.

References

1 Yamashita, S., Koshiya, S., Ishizuka, K. & Kimoto, K. Quantitative annular dark-field imaging of single-layer graphene. *Microscopy* **64**, 143-150, doi:10.1093/jmicro/dfu115 (2015).

2 Martinez, G. T. *et al.* Quantitative STEM normalisation: The importance of the electron flux. *Ultramicroscopy* **159**, 46-58, doi:10.1016/j.ultramic.2015.07.010 (2015).

3 Jones, L., Varambhia, A., Sawada, H. & Nellist, P. D. An optical configuration for fastidious STEM detector calibration and the effect of the objective-lens pre-field. *J. Microscopy-Oxford* (in print), doi:10.1111/jmi.12672 (2018).

4 Kimoto, K. *et al.* Local crystal structure analysis with several picometer precision using scanning transmission electron microscopy. *Ultramicroscopy* **110**, 778-782, doi:10.1016/j.ultramic.2009.11.014 (2010).

5 Kimoto, K., Xie, R.-J., Matsui, Y., Ishizuka, K. & Hirosaki, N. Direct observation of single dopant atom in light-emitting phosphor of β-SiAlON:Eu^{2+}. *Appl. Phys. Lett.* **94**, 041908, doi:10.1063/1.3076110 (2009).

6 Yamashita, S. *et al.* Quantitative annular dark-field imaging of single-layer graphene-II: atomic-resolution image contrast. *Microscopy* **64**, 409-418, doi:10.1093/jmicro/dfv053 (2015).

7 Weickenmeier, A. & Kohl, H. Computation of absorptive form-factors for high-energy electron-diffraction. *Acta Crystallogr. A* **47**, 590-597, doi:10.1107/s0108767391004804 (1991).

8 Kirkland, E. J. *Advanced Computing in Electron Microscopy, Second Ed.* (Springer, 2010).

9 Bird, D. M. & King, Q. A. Absorptive form-factors for high-energy electron-diffraction. *Acta Crystallogr. A* **46**, 202-208, doi:10.1107/s0108767389011906 (1990).
